# Covid-19 vaccine management (Comirnaty and mrna-1273 Moderna) in a teaching hospital in Italy: a short report on the vaccination campaign

**DOI:** 10.1186/s12199-021-01018-z

**Published:** 2021-09-30

**Authors:** Francesca Papini, Niccolò Grassi, Giovanni Guglielmi, Vittorio Gattini, Lucia Rago, Costanza Bisordi, Monica Scateni, Michele Totaro, Alberto Tulipani, Andrea Porretta, Lara Tavoschi, Jacopo Guercini, Grazia Luchini, Silvia Briani, Gaetano Pierpaolo Privitera, Angelo Baggiani

**Affiliations:** 1 Department of Translational Research and the New Technologies in Medicine and Surgery, University of Pisa, Pisa, Italy; 2 The Azienda Ospedaliero Universitaria Pisana, Pisa, Italy

**Keywords:** SARS-CoV-2, mRNA vaccine, Vaccination campaign

## Abstract

**Objectives:**

In this article, we aim to share our experience in the hospital reorganization made to conduct the SARS-CoV-2 vaccination campaign, based on the principles of flexibility and adaptability.

**Study design:**

A descriptive study.

**Methods:**

The data concerning the organization of the vaccination campaign were taken from the operative protocol developed by the hospital dedicated task force, composed by experts in hygiene, public health, occupational medicine, pharmacists, nurses, hospital quality, and disaster managers. Data about the numbers of vaccine administered daily were collected by the Innovation and Development Operative Unit database.

**Results:**

Vaccinations against COVID-19 started across the EU on the 27th of December 2020. The first phase of the vaccination campaign carried out in our hospital was directed to healthcare workers immunization including medical residents, social care operators, administrative staff and technicians, students of medicine, and health professions trainees. The second phase was enlarged to the coverage of extremely fragile subjects. Thanks to the massive employment of healthcare workers and the establishment of dynamic pathways, it was possible to achieve short turnaround times and a large number of doses administered daily, with peaks of 870 vaccines per day. From the 27th of December up to the 14th of March a total of 26,341 doses of Pfizer have been administered. 13,584 were first doses and 12,757 were second doses. From the 4th to the 14th of March, 296 first doses of Moderna were dispensed. It was necessary to implement adequate spaces and areas adopting anti-contagion safety measures: waiting area for subjects to be vaccinated, working rooms for the dilution of the vaccine and the storage of the material, vaccination rooms, post-vaccination observation areas, room for observation, and treatment of any adverse reactions, with an emergency cart available in each working area.

**Conclusions:**

The teaching hospital of Pisa faced the beginning of the immunization campaign readjusting its spaces, planning an adequate hospital vaccination area and providing an organization plan to ensure the achievement of the targets of the campaign. This represented a challenge due to limited vaccine doses supplied and the multisectoral teams of professionals to coordinate in the shortest time and the safest way possible. The organizational model adopted proved to be adequate and therefore exploited also for the second phase aimed to extremely fragile subjects.

Coronavirus disease 2019 (Covid-19) has affected tens of millions of people globally since it was declared a pandemic by the World Health Organization on March 11, 2020 [1, 2].

Considering the current evolution of the pandemic, vaccines against SARS-CoV-2 and their global access are a priority to end the emergency [3].

On 21 December 2020, the European Medicine Agency (EMA) gave formal marketing authorization for the use of mRNA-based COVID-19 vaccine BNT162b2 (Comirnaty) produced by BioNTech and Pfizer for the active immunization in European Union. On 06 January 2021 EMA also authorized mRNA-based COVID-19 vaccine mRNA-1273 (MODERNA), produced by Moderna. The Azienda Ospedaliero-Universitaria Pisana (AOUP) in Pisa is a teaching hospital involved since the beginning of the pandemic in the management of Covid-19 patients. Therefore, the hospital underwent a massive reorganization and reconversion of its spaces and activity to cope with the emergency. The aim of this report is to share our experience in the reorganization of hospital spaces and professionals made to conduct the vaccination campaign in the fastest and safest way possible. Having a large number of operators exposed to SARS-CoV-2, AOUP joined the first phase of the national vaccination campaign against Covid-19. Starting by the 27th of December (EU vaccination day), Comirnaty vaccine was offered to healthcare workers (HCWs), including medical staff, medical residents, social care operators, administrative staff, and technicians. AOUP being a teaching hospital, medical students from the third year onward and health professions trainees were also enlisted.

HCWs who adhered to the vaccination campaign could book on an online regional registration platform a defined time slot for the first dose administration. The centralized regional website automatically scheduled the date for the administration of the second dose.

Phase two started on the 4th of March with the availability of the Moderna vaccine: in this phase, according to the greater risk of death related to Covid-19, priority has been given to extremely fragile individuals, starting with the patients followed by hospital specialists [4].

The limited availability of vaccine doses, the multisectoral team of professionals to coordinate and the reorganization of spaces and logistics were challenges the hospital had to face to ensure the achievement of the targets of the vaccination program in the shortest time and the safest way possible.

To overcome this challenge, the AOUP created a dedicated task force composed by experts in hygiene, public health, occupational medicine, pharmacists, nurses, hospital quality, and disaster managers, who wrote the operative protocol for the vaccine administration.

The planning of the reception, preparation, and transport of the vaccine doses was of uttermost importance to ensure a smooth roll-out of the vaccination campaign [5].

Vaccines requiring a standard cold chain (2/8 °C) followed a distribution model based on a single storage site and a series of second-level territorial hubs. Vaccines requiring an extreme cold chain (− 20/− 70 °C) were delivered directly by the manufacturer to the national hubs identified by the national Ministry of Health.

AOUP is one of the hub hospitals equipped with cooling facilities that can meet the conditions required by Comirnaty and Moderna vaccines [6, 7].

Reception, storage, preparation, and transport of the vaccine doses were performed in the hospital pharmacy under the responsibility of pharmacists and technical operators.

After the check of transport temperature, vaccines were stored (at − 70 °C for Comirnaty and – 20 °C for Moderna) and stored in a safe place.

Hospital pharmacists were in charge of vaccine management, which consists of planning the requested daily vaccine doses, defrosting them (2–8 °C), and packaging the required vials, labeling the defrosted ones and ensuring the process traceability.

The prepared vials were collected and delivered from the Hospital Pharmacy to the Hospital vaccination area just before each vaccination session, where a nurse, supervised by a pharmacist, performed dilution (for vaccines requiring it) and prepared the tray with the doses.

Vaccination lists were printed daily at the beginning of every vaccination session by administrative staff, while nurses verify presence and efficiency of emergency devices and delivery adrenaline syringes in each vaccination room. Emergency carts were available for each vaccination area.

Vaccinees registered for the defined time slots were accepted at the check point set at the facility’s entrance, where a nurse checked body temperature and hand disinfection, assigned a numerical code, and addressed vaccinees to the waiting room, where the call code was rechecked and vaccinees sent to the first vaccination room available. In order to contain the waiting time, vaccinees were requested to fill in advance a form about the medical history which was revised by a doctor directly in the vaccination room.

After collecting medical history, the physician registered the data in the online portal while the vaccine was administered by a nurse. High touch surfaces were disinfected after every vaccination.

After the vaccine administration, vaccinees waited in a post-vaccination observation area, supervised by the healthcare staff for at least 15 min. An intensive care specialist was always promptly available to treat any adverse reaction. A room dedicated to adverse reaction treatment has been set up closely to each observation room. Separate working rooms were set up for the dilution and the storage of the vaccine doses. The routes in the vaccination area were identified by appropriate signs.

At the end of every vaccination session, a nurse archived signed consents, managed stock materials, and surveyed the proper disposal of used vials. Cleaning staff completed ambulatory disinfection.

The organization of the vaccination pathways is depicted in Fig. 1.

**Fig. 1:**
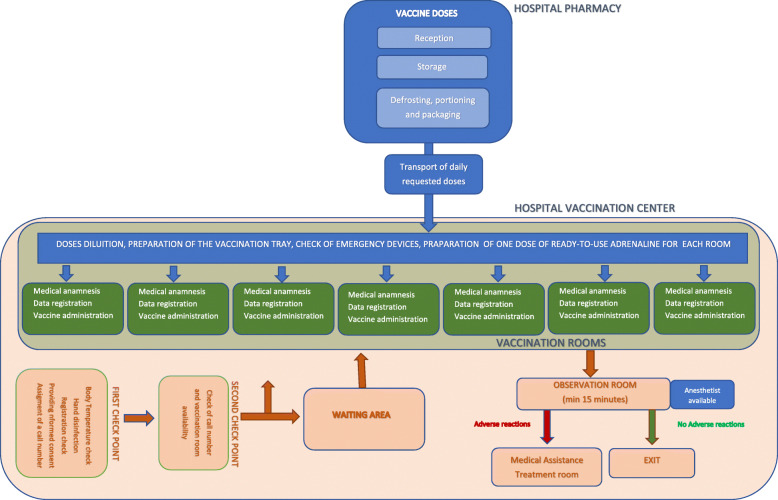
The organization of the vaccination pathways

In order to achieve the assigned goals, it was necessary to deploy a large workforce, with the massive voluntary engagement of HCWs, doctors, nurses, medical residents, pharmacists, and administrative staff.

All the HCWs involved in the vaccine administration were trained to manage any adverse reaction due to the vaccination.

All the records related to vaccine administration (patient data, inoculation site, physician and nurse name, vial’s batch number, and dose number) were collected on a dedicated regional repository.

The organization of the vaccination session was scheduled to provide at least six vaccines per hour for each vaccination room. The organization of the pathways, with anamnesis and medical history collected directly in the vaccination room, made it possible to reduce at the minimum time the permanence in a common waiting area with an increased safety for both patients and operators and to significantly reduce the turnaround times.

Thanks to the workforce employed and to the dynamic pathways planned to reduce the lapse of time between each vaccination, from the 27th of December up to the 14th of March a total of 26,341 doses of Pfizer have been administered. 13,584 were first doses and 12,757 were second doses. From the 4th to the 14th of March, 296 first doses of Moderna were dispensed. The daily amount of vaccines administered depended on the availability of doses and reached peaks of 870 total doses with seven vaccination rooms working.

The vaccinated HCWs were invited to undergo a serological sample for the evaluation of the antibody titer, the results of which are still being collected.

Describing our experience in the initial phases of the vaccination campaign performed in our hospital may help others in the organization of workforces and spaces suitable for an efficient and safe vaccination.

## Data Availability

The datasets used and/or analyzed during the current study are available from the corresponding author on reasonable request

## Abbreviations

SARS-CoV-2: Severe Acute Respiratory Syndrome Coronavirus 2
EMA: European Medicine Agency
AOUP: Azienda Ospedaliero-Universitaria Pisana
HCWs: Healthcare workers
